# Improved Testing of Recent HIV-1 Infections with the BioRad Avidity Assay Compared to the Limiting Antigen Avidity Assay and BED Capture Enzyme Immunoassay: Evaluation Using Reference Sample Panels from the German Seroconverter Cohort

**DOI:** 10.1371/journal.pone.0098038

**Published:** 2014-06-03

**Authors:** Andrea Hauser, Claudia Santos-Hoevener, Karolin Meixenberger, Ruth Zimmermann, Sybille Somogyi, Stefan Fiedler, Alexandra Hofmann, Barbara Bartmeyer, Klaus Jansen, Osamah Hamouda, Norbert Bannert, Claudia Kuecherer

**Affiliations:** 1 Division of HIV and Other Retroviruses, Robert Koch Institute, Berlin, Germany; 2 Division of HIV/AIDS, STI and Blood-borne Infections, Robert Koch Institute, Berlin, Germany; University of Cape Town, South Africa

## Abstract

**Background:**

The variety and limitations of current laboratory methods for estimating HIV-incidence has driven attempts to improve and standardize the performance of serological ‘Tests for Recent HIV-Infections’ (TRI). Primary and follow-up HIV-1 positive plasma samples from individuals with well-defined dates of infection collected as part of the German Seroconverter Cohort provided specimens highly suitable for use in comparing the performance of three TRIs: the AWARE™ BED™ EIA HIV-1 Incidence test (BED-CEIA), Genetic systems HIV-1/HIV-2 Plus O EIA antibody avidity-based assay (BioRad Avidity) and Sedia™ HIV-1 LAg Avidity EIA (LAg Avidity).

**Methods:**

The evaluation panel included 180 specimens: 44 from antiretroviral (ARV)-naïve individuals with recently acquired HIV-infection (≤130 days; 25 B and 19 non-B subtypes) and 136 from long-term (>12 months) infected individuals [101 ARV-naïve subtype B, 16 non-B subtypes, 14 ARV-treated individuals, 5 slow progressors (SLP)].

**Results:**

For long-term infected, ARV-naïve individuals the false recent rates (FRR) of both the BioRad and LAg Avidity assays were 2% (2/101 for subtype B) and 6% (1/16 for subtype ‘non-B’), while the FRR of the BED-CEIA was 7% (7/101 for subtype B) and 25% (4/16 for subtype ‘non-B’) (all *p>*0.05). Misclassification of ARV-treated individuals and SLP was rare by LAg (1/14, 0/5) and BioRad Avidity assays (2/14, 1/5) but more frequent by BED-CEIA (5/14, 3/5). Among recently-infected individuals (subtype B), 60% (15/25) were correctly classified by BED-CEIA, 88% (22/25) by BioRad Avidity and significantly fewer by LAg (48%, 12/25) compared to BioRad Avidity (*p* = 0.005) with a higher true-recency rate among non-B infections for all assays.

**Conclusions:**

This study using well-characterized specimens demonstrated lower FRRs for both avidity methods than with the BED-CEIA. For recently infected individuals the BioRad Avidity assay was shown to give the most accurate results.

## Introduction

Monitoring the HIV-epidemic and identifying populations amongst whom HIV is spreading is critical for public health services, allowing them to identify groups at risk and target preventive interventions effectively. Reliable methods to estimate HIV incidence in cross-sectional surveys are therefore crucially important. Over the past fifteen years, a variety of serological assays able to differentiate recently acquired from established HIV-1 infections have been developed [1]–[4]. Most of these ‘Tests for Recent (HIV) Infections’ (TRI) are based on the fact that evolution and maturation of HIV-1 specific antibodies occur within the first two years after seroconversion [5], [6]. Accordingly, incidence assays differentiate between recently acquired and long-term infections based on i) the increase in antibody titer [7]–[9], ii) the increase in the proportion of HIV-1 specific immunoglobulin G (IgG) antibodies relative to total IgG [10], iii) the increase of antibody avidity [11]–[15] or iv) a combination of these markers [6], [16]–[20]. Classification of individuals as either recently or long-term infected is finally determined by the quantitative output relative to a defined cut off.

For many years, the BED-capture-enzyme immunoassay (BED-CEIA) [10] was the most commonly used incidence assay for HIV-surveillance, both in Germany and worldwide [21]–[25]. However, reports of substantial over-estimation of incidence due to the misclassification of established infections as recently acquired infections dampened the acceptance and use of the BED-CEIA [26], [27]. Although formula-based adjustment for BED-CEIA estimates have been proposed [28]–[30], these limitations prompted the search for novel assays with improved accuracy.

In 2010 the performance of a new avidity-based assay using a modified version of the BioRad HIV-1/2 Plus O protocol (BioRad Avidity) was presented by Masciotra *et al.* 2010 [13] showing improvements in test accuracy [31], [32].

Furthermore, in 2010 two new avidity-based assays using multi-subtype gp41 recombinant protein in a two-well and a novel single-well format were described, the latter of which becoming commercially available as Sedia TM HIV-1 LAg Avidity EIA (LAg Avidity) [14], [33].

However, currently available incidence assays continue to be challenged by the variability of immune responses among infected persons. One of the main problems is the identification of ‘false recent’ infections as a result of low HIV-antibody titer or low binding affinity. This type of misclassification occurred particularly for long-term infected individuals on antiretroviral (ARV) treatment, individuals with advanced AIDS progression and for elite controllers [4], [31], [34]–[36]. Additionally, the accuracy of the assays was found to vary depending on the viral subtype [37]–[39].

The ‘Consortium for the Evaluation and Performance of HIV Incidence Assays’ (CEPHIA) [40], a collaboration of international public health professionals and scientists founded by the Bill & Melinda Gates Foundation, is currently evaluating of the most commonly used incidence assays (‘candidate’ assays) [41] in order to identify a test that is quick, inexpensive, easy-to-use, valid, robust, precise and provides a reliable standard method or algorithm for estimating incidence [40]. Test performance focuses on two interacting test parameters that jointly specify the test characteristics: the mean duration of recency (MDR) as the average time that an individual is classified as recently infected (proposed to be 4–12 months) and the false recent rate (FRR) - characterizing the frequency of misclassified long-term infections as recent infection - which should be <2% [31], [36], [41]. To allow comparisons of test evaluations CEPHIA established a specimen repository comprising of recent and long-term (>12 months) infection specimens, as well as ‘challenge specimens’ that include samples from elite controllers, ARV-treated/suppressed individuals and non-B subtypes of HIV-1 [42], [43].

The German HIV-1 Seroconverter study - a national multicenter long-term observational open cohort study running since 1997 - comprises longitudinal HIV-1 positive plasma specimens from individuals with well-defined periods of infection. These specimens, precisely characterized in terms of duration of infection and detailed course of ARV-treatment, offer the opportunity to evaluate the performance of selected ‘candidate’ assays. In the present study, the performance of the BioRad Avidity and the commercially available LAg Avidity assays was compared to that of BED-CEIA with regard to the classification of recent infections and FRR.

## Materials and Methods

### Ethics Statement

Signed informed consent is obtained from all subjects prior to enrolment. The study is approved by the ethical committee of Charité- Universitätsmedizin Berlin, Germany.

### Evaluation Panel

All specimens included in the evaluation panel of the present study were primary or follow- up samples collected within the German HIV-1 Seroconverter (SC) Cohort [44]. The dates of infection are well-defined by the following documented laboratory test results:

(1) Detectable HIV-RNA plus negative ELISA OR a reactive ELISA plus negative or indeterminate immunoblot as evidence of an ongoing but incomplete seroconversion (‘acute SC’); completion of seroconversion is subsequently confirmed during follow-up within six months. For these patients the date of infection is defined as the blood sampling date for the first reactive test. (2) A last negative and a first positive documented HIV-antibody test result (‘documented SC’) are available. The date of infection is calculated as the arithmetic mean of both test dates. For inclusion into the present work only documented SC were included for whom the negative and positive HIV-antibody test results were available with a maximal time interval of 90 days.

The evaluation panel comprised sets of ‘recent infections’, ‘long-term infections’ and a ‘challenge specimens’. All specimens chosen for the ‘recent infections’ set were immunoblot positive and had been collected ≥14 days and ≤130 days after the defined date of infection. Samples included in the ‘long-term infection’ and ‘challenge specimen’ sets were selected from collections ≥52 weeks after the defined date of infection. To avoid individual-specific bias in assay performance only one specimen per HIV-positive patient was analyzed. In total, the evaluation panel was made up of 180 cross-sectional samples. All specimens included in the ‘recent’ set (n = 44; 25 B subtypes and 19 non-B subtypes) and the ‘long-term infections’ set (n = 117; 101 B subtypes and 16 non-B subtypes) were collected from ARV-naïve individuals.

The ‘challenge specimens’ set (n = 19) comprised a subset of 14 plasma samples from ARV-treated individuals with either CDC status A or B and CD4 cell counts >350 cells/µl (n = 8) or with advanced disease progression as characterized by: CDC status C and CD4 cell counts ≤350 cells/µl (n = 6). A second subset of the ‘challenge specimens’ consisted of five plasma samples from ARV-naïve individuals with slow disease progression, defined by CD4 cell counts (at least 3 available) that did not fall below 500 cells/µl for at least 8 years of infection (Table 1).

**Table 1:** Characteristics of evaluation panel.

Characteristics		Recent infections (n = 44)		Long-term infections (n = 117)		“Challenge specimens” (n = 19)			
		Subtype B	Subtype „non-B“	Subtype B	Subtype „non-B“	ARV-treated		Slow progressors	
**Number of specimen**		**25**	**19**	**101**	**16**	**14**		**5**	
Age	Median (IQR)	33 (29–39)	40 (31–47)	34 (30–39)	36 (27–44)	35 (26–39)		32 (24–33)	
Sex	Male	25 (100%)	12 (63%)	100 (99%)	9 (56%)	14 (100%)		5 (100%)	
	Female	-	7 (37%)	1 (1%)	7 (44%)	-		-	
Category of SC	Acute SC	25 (100%)	18 (95%)	100 (99%)	14 (88%)	8 (57%)		2 (40%)	
	Documented SC	0	1 (5%)	1 (1%)	2 (12%)	6 (34%)		3 (60%)	
Duration of infection (IQR)		62 d (42–89)	35 d (28–54)	122 wk (83–168)	100 wk (65–179)	308 wk (149–400)		447 wk (438–452)	
Risk groups	MSM	25 (100%)	5 (26%)	94 (93%)	5 (31%)	14 (100%)		4 (80%)	
	Hetero	-	10 (52%)	3 (3%)	10 (62%)	-		-	
	IDUs	-	2 (11%)	1 (1%)	1 (6%)	-		-	
	Unknown/other	-	2 (11%)	3 (3%)	-	-		1 (20%)	
CD4 cell count	Cells/µl (IQR)	561 (430–678)	511 (276–787)	409 (314–538)	438 (268–567)	set 1 (n = 8): 484 (403–595)		714 (601–967)	
						set 2 (n = 6): 261 (257–299)			
Subtype		B	A1,C,CRF01_AE,	B	A1,C,CRF01_AE,	B; 1x not analyzed		B	
			D,F1,CRF02_AG		D,G,CRF02_AG				

IQR = Interquartile range, SC = seroconverter, d = days, wk = weeks.

The ‘recent infections’ set was mainly selected from those ‘acute SC’ individuals with the most precise dates of infection: 100% (25/25) in the subset of subtype B samples and 95% (18/19) in the non-B samples. In the ‘long-term infections’ set 99% (100/101) and 88% (14/16) of follow-up specimen were obtained from ‘acute SC’ for the subtype B and non-B subsets, respectively. The ‘challenge specimens’ set was composed of 57% (8/14) and 40% (2/5) ‘acute SC’ (long-term infected ARV-treated individuals and slow progressors, respectively) (table 1). The FRR was calculated from the number of specimen falsely classified as ‘recent’ within the ‘long-term infections’ or ‘challenge specimens’ sets.

Within the German SC Cohort, patient characteristics such as demographic (including sex and age at enrolment), clinical status (including CDC-status and current ARV regimens) and laboratory data (including viral load, CD4+ and CD8+ cell counts) are provided with the samples. Longitudinal plasma samples and clinical information are collected in yearly follow-ups. Signed informed consent is obtained from all subjects prior to enrolment. The study is approved by the ethical committee of Charité- Universitätsmedizin Berlin, Germany.

The REGA HIV-1 subtyping tool [42], [43], [45] was used to subtype HIV-1 based on the *pol* population sequences determined during genotypic resistance monitoring (Viroseq HIV-1 Genotyping System version 2, Abbott, Wiesbaden, Germany).

### Methodology

All specimens were analyzed using the three HIV-1 incidence assays BED-CEIA, BioRad Avidity and LAg Avidity according to the recommended protocols.

The commercially available BED-CEIA (AWARE™ BED™ EIA; Calypte Biomedical Corporation, Lake Oswego, OR, USA) is a single well incidence assay. The proportion of HIV-1 specific anti-gp41-IgG compared to total IgG in the sample is determined by a calibrator-normalized OD value (ODn). ODn values below the cut off of 0.8 are classified as recent infections. The MDR as described in the manufacturerś instructions is 155 days.

For the BioRad Avidity assay modifications of the commercial Genetic Systems HIV-1/HIV-2 Plus O EIA (Bio-Rad Laboratories, Redmond, WA, USA) were made. In the two-well avidity assay specimens diluted 1∶10 are initially incubated 30 minutes at 4°C and were then treated in parallel with either 0.1 M DEA dissociation agent or wash buffer. An avidity index (AI) is calculated from both OD values (OD (DEA)/OD (wash buffer) x100) for each sample. Specimens with an AI below 30% are classified as ‘recent infections’ [13]. One recent and prevalent sample with known duration of infection each (three and 58 weeks of infection, respectively) are used as internal test controls. The MDR according to Masciotra and Owen (personal communication) is 220 days for infections with subtype B or 209 days for infections with non-B subtypes.

The LAg Avidity (Sedia™ HIV-1 LAg Avidity EIA; Sedia Biosciences Corporation, Portland, OR, USA) is a new commercially available antibody avidity-based single well incidence assay. Patientś antibodies are allowed to bind to recombinant proteins containing the HIV-1 immune dominant region (IDR) of gp41 (coating antigen). Using 0.1 M citrate buffer as a dissociation agent, the antibody avidity is measured as an ODn value adjusted by calibrator and controls. According to the 2013 manual specimens with an ODn value below 1.5 are classified as ‘recent’ and the respective MDR is 130 days.

To identify significant differences between the median quantitative assay outputs for specimens from the ‘recent’ and ‘long-term infections’ set, statistical analyses were carried out using the non-parametric Mann-Whitney-U test (IBM SPSS Statistics 20; SPSS Inc., Chicago, Illinois, USA). The independence of categorical variables was analyzed using the two-tailed Fisher’s exact test that can be applied to small sample sizes (GraphPad QuickCalcs: http://www.graphpad.com/quickcalcs/contingency1.cfm). Only p-values below 0.05 were considered to be significant.

## Results

### Characteristics of the Evaluation Panel

Characteristics of the ‘recent’, ‘long-term’ and ‘challenge’ sets used for evaluation are shown in Table 1.

Among the subset of ‘recent HIV-1 non-B infections’, HIV subtypes A1 (n = 5), C (n = 4), CRF01_AE (n = 3), CRF02_AG (n = 4), D (n = 1) and F1 (n = 2) were identified. Subtypes A1 (n = 3), C (n = 4), CRF01_AE (n = 1), CRF02_AG (n = 5), D (n = 1) and G (n = 2) were identified in the ‘long-term non-B infections’ subset. Slow progressors and ARV-treated individuals were all infected with subtype B, although no sequence was available for one patient and the subtype remained unknown (Table 1).

**Figure 1:**
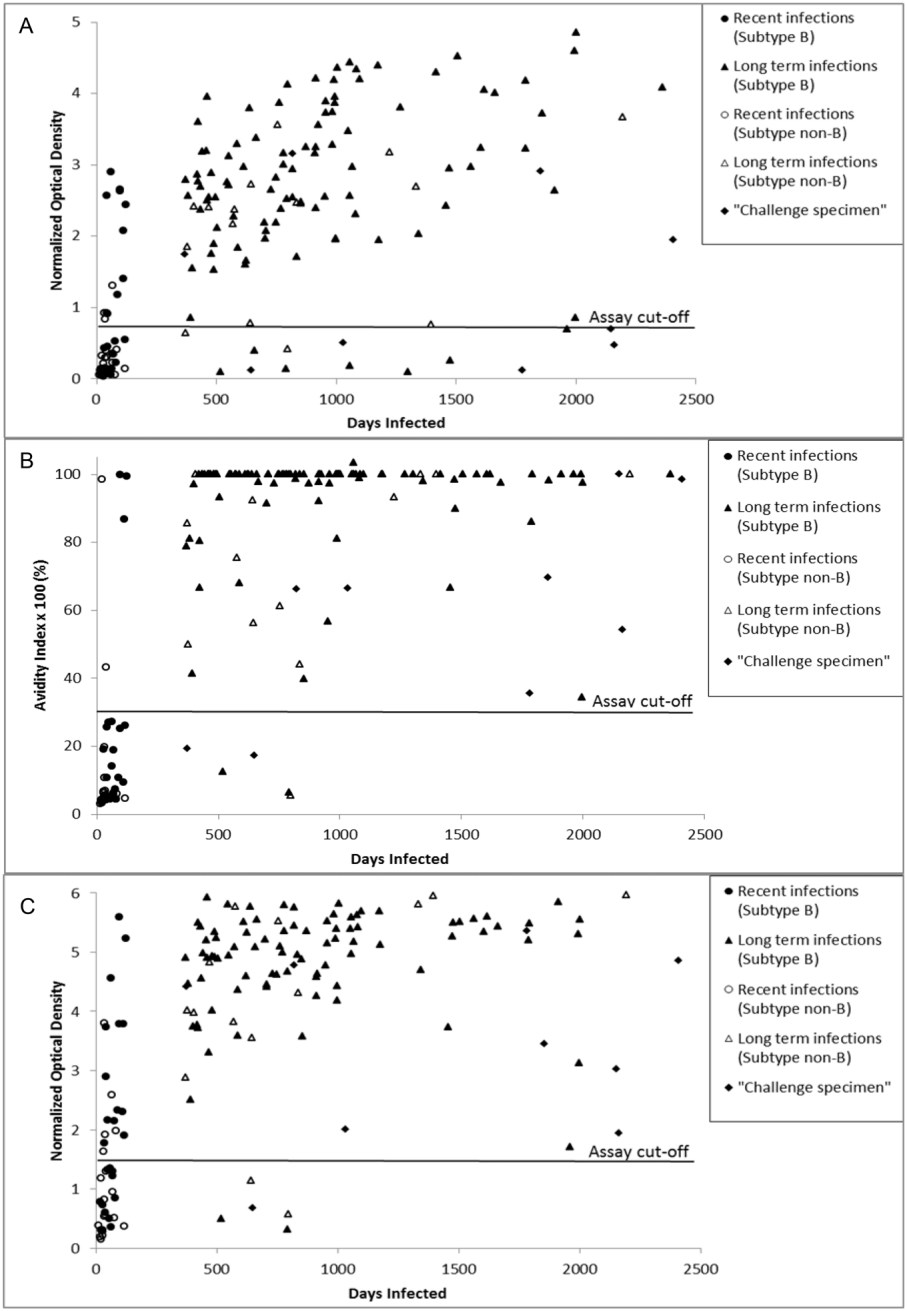
BED-CEIA, Bio-Rad Avidity and Lag-Avidity results according to duration of infection. Samples from the evaluation panel were analyzed using the BED capture enzyme immunoassay (A), Bio-Rad Avidity assay (B) and LAg-Avidity enzyme immunoassay (C). Respective assay cut-offs are indicated by the black line. Data are shown for subsets of the evaluation panel.

The median BED-CEIA ODn value was significant lower in ‘recent infections’ set than in the ‘long-term infections’ set (0.3 (IQR 0.1–0.9) vs. 2.7 (IQR 1.9–3.6); p<0.001; Figure 1a). The median BioRad AI was also significantly lower for ‘recent infections’ than for ‘long-term infections’ (6.2% (IQR 4.6–19) vs. 100% (IQR 86–100); p<0.001; Figure 1b). In addition, the median LAg Avidity ODn value was significantly lower in the ‘recent infections’ set than in the ‘long-term infections’ set (1.3 (IQR 0.5–2.2) vs. 5.1 (IQR 4.4–5.6); p<0.001; Figure 1c).

### Comparison of True and False Recent Rates between the Three Incidence Tests

**Figure 2:**
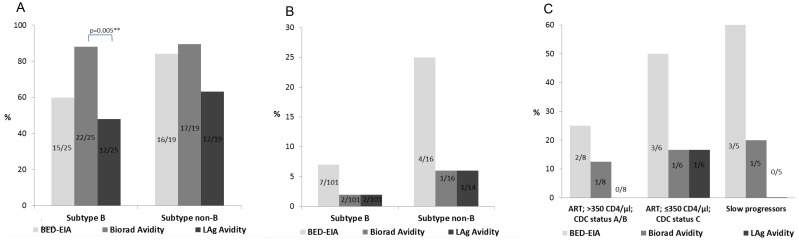
Comparison of BED-CEIA, Bio-Rad and LAg-Avidity assay results according to subtype, clinical stage and disease progression. True recent ratios within the ‘recent infections set’ subtype B and ‘non-B’ (A). False recent ratios among long-term subtype B and ‘non-B’ infected, ARV-naïve individuals (B). Misclassification in the ‘challenge specimens’ set (C). Only significant p-values (<0.05) of pairwise comparisons were indicated.

Among individuals with recently acquired subtype B infection 60% (15/25) were correctly classified by BED-CEIA, 88% (22/25) by BioRad Avidity and 48% (12/25) by LAg Avidity. The proportion of correctly classified recent samples was significantly higher by BioRad Avidity compared to LAg Avidity (*p*<0.01) and tended to be higher for BED-CIA (*p* = 0.05) whereas the differences between BED-CEIA and LAg Avidity (*p* = 0.57) were less pronounced. Correct recency rates among individuals with HIV-1 ‘non-B’ infections were: 84% (16/19) by BED-CEIA, 89% (17/19) by BioRad Avidity and 63% (12/19) by LAg Avidity with none of the differences being statistically significant (all *p*>0.05) (Figure 2a).

The FRRs in the subsets of ‘long-term infections’ were 7% (subtype B) and 25% (‘non-B’) for the BED-CEIA and 2% (subtype B) and 6% (‘non-B’) for both avidity assays BioRad and LAg Avidity (Figure 2b). Although misclassification of ‘challenge specimens’ occurred at most once using the LAg Avidity (1/8, 1/6, 0/5) and BioRad Avidity (1/8, 1/6, 1/5) assays within the subsets of long-term infected, ARV-treated individuals (CD4 cell counts ≤ or >350 cells/µl) and slow progressors, it occurred more frequently by BED-CEIA (2/8, 3/6, 3/5). The overall FRRs (‘long-term infections’ plus ‘challenge specimen’) for BED-CEIA (14%), BioRad (4.4%) and LAg Avidity (3.7%) were significantly higher for BED-CEIA compared to BioRad (*p = *0.01) and LAg Avidity (*p = *0.005), but pairwise comparisons between tests for all subsets of ‘long-term infections’ (Figure 1b) and ‘challenge specimens’ (Figure 2c) did not reach statistical significance (all *p*>0.05).

#### Misclassification of samples by the three incidence assays

**Figure 3:**
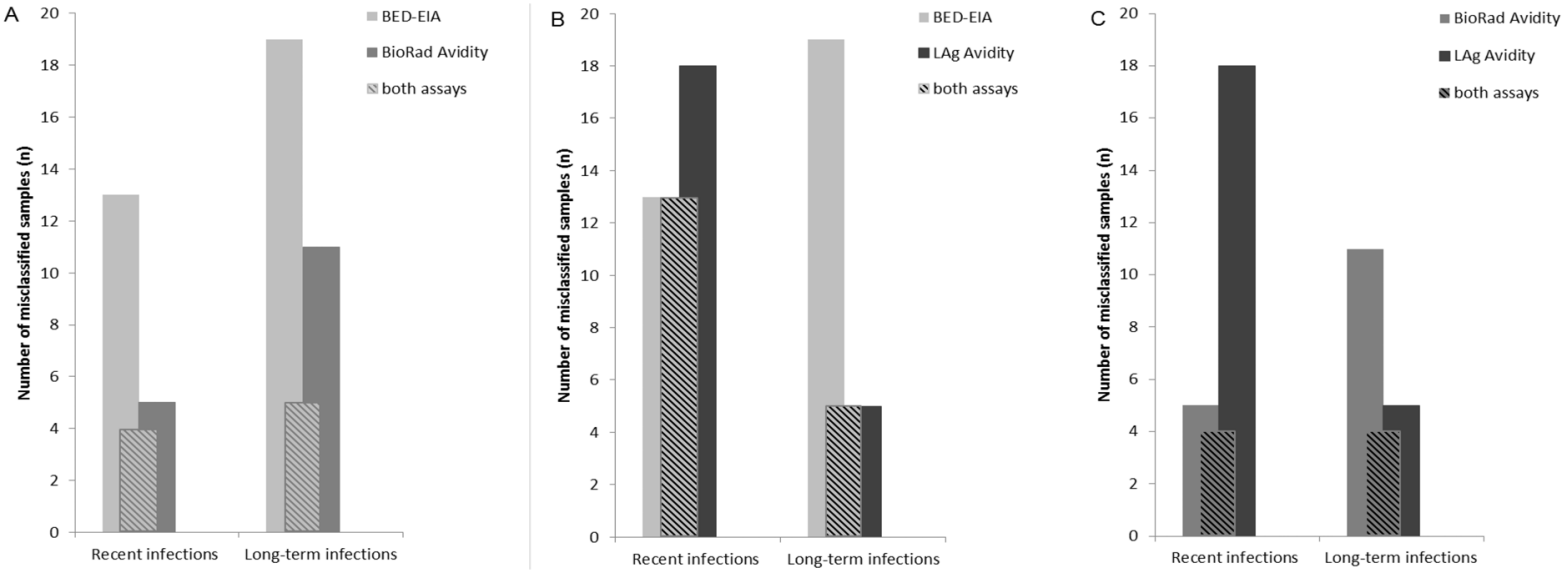
Number of samples in the set of recent (n = 44) and long-term infections (incl. ‘challenge specimens’; n = 136) misclassified by two incidence assays. Misclassified samples (A) by the BED-CEIA, BioRad Avidity and by both assays (B) by the BED-CEIA, LAg Avidity and by both assays and (C) by BioRad Avidity, LAg Avidity and by both assays.

Some of the samples misclassified by one of the three assays were also misclassified by one or both of the other two incidence tests (Figure 3). In particular, all samples misclassified by BED-CEIA in the ‘recent infections’ set (n = 13/44) were also misclassified by LAg Avidity (18/44; intersection of n = 13 samples) and all samples classified as ‘false recent’ by LAg Avidity in the ‘long-term’ and ‘challenge specimens’ set (n = 5/136) were also misclassified by BED-CEIA (19/136; intersection of n = 5 samples; Figure 3B). In contrast, of the 5/44 and 13/44 “recent” samples misclassified by the BioRad Avidity and BED-CEIA assays respectively, four were commonly misclassified, and of the 11/136 and 19/136 (respectively) “long-term” infection samples misclassified, five were the same (Figure 3A). Similarly, the LAg Avidity assay, with 18/44 “recent” and 5/136 “long-term” infection misclassified samples had four in common with the BioRad Avidity assay for each set (Figure 3C).

False recent classifications within the subset of ‘long-term non-B infections’ could be assigned to different subtypes: 2/3 for A1, 1/1 for D and 1/4 for C by BED-CEIA, as well as 1/3 for A1 and 1/1 for D by LAg Avidity. The only ‘non-B’ subtype sample giving a false recent classification by the BioRad Avidity assay was shown to be a subtype D isolate that was also misclassified by BED-CEIA and LAg Avidity (data not shown).

## Discussion

FRR and MDR are considered to be important parameters for the evaluation of incidence assays [4], [31], [41], [46]. While MDRs were defined by the manufacturerś instructions (BED-CEIA and LAg Avidity) or provided with the protocol (BioRad Avidity) [13], the FRR was calculated for the three incidence assays BED-CEIA, BioRad Avidity and LAg Avidity using primary and follow-up samples with known dates of infection selected from the German Seroconverter Cohort.

Compared to the BED-CEIA the two antibody avidity-based assays (LAg Avidity and BioRad Avidity) generally gave significantly lower proportions of false recent classifications for long-term infected individuals and therefore lower FRRs. High FRRs from the BED-CEIA, in particular for individuals undergoing ARV treatment, those with advanced progression to AIDS and for elite controllers have also been described previously [47], [48]. Since the BED-CEIA is based on the proportion of HIV-specific IgG antibodies relative to the total quantity of IgG, individuals with viral suppression (elite controllers or ARV treated) or waning antibodies due to advanced HIV progression (in our study CDC status C) are at risk of being misclassified as recent. In contrast, antibody avidity depends on the degree of antibody maturation, which in turn is directly correlated with duration of infection [2]. Samples with decreased antibody content, as reported for individuals with viral suppression and advanced disease, were correctly classified more often using the avidity assays, resulting in lower FRRs than the BED-CEIA [32], [36], [49]. However, factors such as recent sample collection, viral suppression and lower CD4+ cell counts were recently reported to be associated with false recent classification using the LAg Avidity [50].

Likewise, high FRRs generated by BED-CEIA for individuals with ‘non-B’ infections have been described previously and calculating and adapting the MDR for each ‘non-B’ subtype by generating more data was suggested [39].

In our study, the FRRs by BED-CEIA within the group of immunosuppressed individuals (‘challenge specimens’ subset) and the subset of ‘non-B infections’ were indeed higher compared to both avidity assays, although the differences did not reach significance for the respective subsets. The misclassification of samples from subtype D infected individuals (as occurred in our study using all three assays) was also recently reported for BioRad and LAg Avidity assays [50], [51] and was reported to be due to a weak initial antibody response to HIV infection that is maintained over time. However, the number of samples in the subsets of non-B infections and the ‘challenge specimen’ set is far too small to power statistical comparison.

In the set of ‘recent infections’, the BioRad Avidity assay performed best for subtype B infected individuals, giving a significantly higher true recent rate of 88% compared to the relatively low true recent rate of LAg Avidity (48%). Based on the previous LAg Avidity cut-off (ODn = 1.0) and MDR (141 days) as provided by the manufacturer in 2012, an additional four samples (134–141 days of infection, subtype B) would be included in the ‘recent infections’ set resulting in a significantly lower true recent rate for the LAg Avidity assay (31%) compared to those for the Bio Rad Avidity assay (86%, *p* = 0.0001) and BED-CEIA (62%, *p* = 0.03) [52]. The re-adjustment of the LAg Avidity ODn cut-off in 2013 (raising it from 1.0 to 1.5 based on CEPHIA evaluations) improved the performance to give a true recent rate of 48%, although this is still low compared to the BioRad Avidity assay. The increase in cut-off was accompanied by a decrease in the MDR from 141 to 130 days (4.7 to 4.3 months). This very short MDR is further from the proposed MDR of 4–12 months [36].

For the LAg Avidity assay the ratio for correct ‘recent’ classifications in the ‘recent infections’ set was very low (48%) while the ratio for correct classification in the ‘long-term infections’ set was high (98%). For the BED-CEIA the correct ‘recent’ classifications ratio was also low with 60% and far below published data of 82% (previously termed ‘sensitivity’, using the terminology for individual diagnostics) [10]. In this context, it is remarkable that the BED-CEIA and LAg Avidity assays often misclassified the same specimens. In contrast, samples misclassified by BioRad Avidity assay coincided less with those misclassified by BED-CEIA and LAg Avidity. This might be due to the fact that both the BED-CEIA and the LAg Avidity assay are based on gp41 epitopes whereas the antigens used in the BioRad Avidity assay bind to a broader spectrum of the individualś antibodies (IgM, IgG) and might therefore react differently. It is therefore not recommended to use the BED-CEIA and LAg-Avidity assay together in multi-assay algorithms [53]; the use of BioRad Avidity in combination with BED-CEIA or Lag Avidity would be the better choice.

Certainly, FRR is an important parameter, in particular in countries with a low prevalence of HIV, and it is important to limit the misclassification of long-term infections as recent. However, in our opinion, the correct identification of recent samples with the aim of also reducing the number of false long-term samples should not be ignored in an effort to minimize the FRR. In Germany, where the testing for recent infections is based on the analysis of specimens from newly diagnosed individuals, it is of great importance to identify those individuals with recently acquired infection amongst the newly diagnosed to avoid underestimating the rate of new infections as well as overestimating by a high FRR.

One limitation of our study is the relatively low number of samples in the subsets of ‘recent infections’, of ‘non-B infections’ and of the ‘challenge specimens’. This was caused by restricting samples to those from individuals with very well-documented seroconversions and by the low prevalence of ‘non-B’ subtypes and long-term non-progressors in the German SC cohort. However, despite being limited in size, results obtained with this study agree with previous reports and have shown significant differences within the subset of “recent subtype B infections”.

The tentative outcome is nevertheless an indication that among long-term infected ARV naive individuals, both avidity assays delivered lower FRRs than the BED-CEIA. Of the two avidity methods, BioRad Avidity was best at identifying truly recent infections, with a FRR similar to the LAg Avidity. However, although the new 2013 cut-offs improved the LAg Avidity assay, it may still be underestimated the true proportion of recent infections in newly diagnosed individuals. To date, a thorough evaluation of the LAg Avidity and BioRad Avidity assays for use in incidence estimation is still pending and additional studies that include samples with less than one year of infection should be carried out.
